# Isolation of Polyphenols from Aqueous Extract of the Halophyte *Salicornia ramosissima*

**DOI:** 10.3390/molecules29010220

**Published:** 2023-12-31

**Authors:** Malthe Fredsgaard, Job Tchoumtchoua, Stephan Kohnen, Tanmay Chaturvedi, Mette Hedegaard Thomsen

**Affiliations:** 1 AAU Energy, Aalborg University, 6700 Esbjerg, Denmark; mfre@energy.aau.dk (M.F.); tac@energy.aau.dk (T.C.); 2 Biomass Valorisation Platform, CELABOR Scrl, 4650 Herve, Belgium; job.tchoumtchoua@celabor.be (J.T.); stephane.kohnen@celabor.be (S.K.)

**Keywords:** *Salicornia ramosissima*, isolation, purification, polyphenols, liquid–liquid extraction, resin adsorption, membrane filtration, centrifugal partition chromatography

## Abstract

Polyphenols from residual non-food grade *Salicornia ramosissima* have health-promoting effects in feed, food, or nutraceutical applications. Therefore, the isolation of polyphenols is of interest from a series of environmentally friendly isolation methods with recyclable solvents. The isolation of polyphenols from non-food grade *S. ramosissima* was investigated using sequential membrane filtration with and without acid pretreatment, liquid–liquid extraction, resin adsorption, and centrifugal partition chromatography (CPC); analyzed by the Folin–Ciocalteu assay for total polyphenols; and finally analyzed using UPLC-TQMS in negative ion-spray mode for detection of 14 polyphenols. Sequential membrane filtration and acid hydrolysis indicated the polyphenols forming complexes with other compounds, retaining the polyphenols in the retentate fraction of large molecular weight cut-off membrane sizes. Conventional liquid–liquid extraction using sequential ethyl acetate and n-butanol showed most polyphenols were extracted, apart from chlorogenic acids, indicating a low isolation efficiency of higher polarity polyphenols. Analysis of the extract after resin adsorption by Amberlite XAD-4 resin showed high efficiency for separation, with 100% of polyphenols adsorbed to the resin after 13 bed volumes and 96.7% eluted from the resin using ethanol. CPC fractionations were performed to fractionate the concentrated extract after resin adsorption. CPC fractionations of the 14 polyphenols were performed using an organic or aqueous phase as a mobile phase. Depending on the mobile phase, different compounds were isolated in a high concentration. Using these easily scalable methods, it was possible to comprehensively study the polyphenols of interest from *S. ramosissima* and their isolation mechanics. This study will potentially lead the way for the large-scale isolation of polyphenols from *S. ramosissima* and other complex halophytes. The compounds of the highest concentration after CPC fractionation were isoquercitrin and hyperoside (155.27 mg/g), chlorogenic acid (85.54 mg/g), cryptochlorogenic acid (101.50 mg/g), and protocatechuic acid (398.67 mg/g), and further isolation using CPC could potentially yield novel polyphenol nutraceuticals.

## 1. Introduction

A circular biorefinery approach can fully utilize the edible herb using the agricultural residual non-food grade part of the salt-tolerant halophyte *Salicornia ramosissima*. Due to increased consumer awareness of ethically sourced bioderived nutraceuticals, toxic, flammable, costly, and volatile solvents should be avoided in future extraction and isolation applications [1,2]. Biorefining halophytes for their high-value bioactive compounds can obtain a secondary benefit, namely the bioremediation of saline soils. As the obligatory halophytic plants can extract salts from the soils and contain them, removing halophytes cultivated on salt-affected soil and the following downstream process can remove large quantities of salt [3]. *S. ramosissima* is biomass with limited use after cultivation as it is recalcitrant and high in salts, which makes it unsuitable for feed application, incineration, composting, and soil amendments. *S. ramosissima* has been proven to contain various polyphenols, each with potential health benefits when introduced in food, feed, or pharmaceuticals [1,4,5,6]. Many phenolic acids have additional hydroxyl groups prone to deprotonate at pH > pKa after the primary deprotonation of the carboxyl group [2,7]. Deprotonation of phenolic acids, e.g., ferulic acid to ferulate, will increase the ionic strength and make the compounds more hydrophilic. This effect can be manipulated in liquid–liquid extractions, and protonation by lowering pH could potentially increase the mass transfer of polyphenols into an organic, non-water-soluble solvent. Surget et al. proved liquid–liquid extraction to be a feasible method for isolating flavonoids from *S. ramosissima* measured qualitatively by liquid chromatography (LC) triple quadrupole mass spectrometer (TQ-MS) in negative ion mode, with the method based on work by Stiger-Pouvreau [8,9]. The yields of individual polyphenols from the liquid–liquid extraction by Surget were not reported. Polyphenols have previously been isolated and fractionated from aqueous extracts by membrane filtration [10,11,12], resin adsorption [13,14,15], liquid–liquid extraction [8,9,16], preparative HPLC [17,18], centrifugal partition chromatography (CPC) [19,20,21], and supercritical CO2 extraction [22,23].

Conidi et al. isolated the disaccharide flavonoids narirutin, naringin, hesperidin, and neohesperidin from bergamot juice using 100 kDa ultrafiltration to remove suspended solids and submitted to 1000 Da, 750 Da, and 450 Da membranes in parallel. The 450 Da membrane retained most flavonoids and molecules in the 450–100,000 Da range. Sugars and organic acids, however, were found in the permeate [10]. However, membrane filtration can pose difficulty in complex solutions and suspensions. Polyphenols and proteins are found in all plants but can form covalent bonds in protein–polyphenol complexes [24,25]. The metabolic and chemical mechanisms responsible for the interaction between proteins and polyphenols are not yet described in the literature. However, hydrolysis of the bonds could yield higher recovery of the polyphenols and potentially be utilized in physical separation methods, such as membrane filtration [24].

Zadeh and Zeppa investigated the adsorption kinetics of a polyphenol-enriched extract on three Amberlite resins, namely XAD-4, XAD-7, and XAD-16. They found a correlation between the resin structure and surface area with the concentration of polyphenols adsorbed on the resin. XAD-4 and XAD-16, both hydrophobic and polyaromatic, showed the best adsorption capabilities due to the π-π conjugates forming between resin and polyphenols [13]. XAD-4 and XAD-16 being the superior resins for polyphenol adsorption has also been proven by Scordino et al. [26,27], Pinto et al. [28], Kaleh and Geißen [29], and Grohmann et al. [30].

One-dimensional liquid chromatography, preparative HPLC, and two-dimensional liquid chromatography (CPC), are preparative methods of compound isolation by non-polar and polar molecular affinity interactions. CPC is superior to preparative HPLC in scale-up method development, cost efficiency, solvent recycling, targeting small molecules in complex mixtures, the possibility of stationary phase gradient, and column maintenance [31]. Using CPC, Destandau et al. isolated the flavonoid isomers nepetin and isorhamnetin from *Anvillea radiata* aerial parts using a gradient Arizona solvent system [21].

The aim of this study was to examine the isolation of polyphenols from *S. ramossissima*. Hence, two extraction methodologies, water-based Soxhlet extraction and dynamic sub-critical water extraction (DSCE), were applied to produce extracts that were further processed by three fractionation approaches: membrane filtration, liquid–liquid extraction (water-based Soxhlet extraction), and non-polar resin adsorption (for DSCE). Finally, the novel high throughput preparative CPC method was applied to isolate high-added value individual polyphenols.

The extracts and isolation methods were applied by Aalborg University and Celabor as part of the Horizon 2020 project AQUACOMBINE. The extracts produced by Soxhlet extraction were made by scientists at Aalborg University, and the extracts produced by DSCE were made by scientists at Celabor. All data were produced by scientists from Aalborg University and Celabor with their individually available methods.

## 2. Results and Discussion

### 2.1. Extracts

The extraction methods used for this study of the isolation of polyphenols from *S. ramosissima* are chosen as they are shown to extract large amounts of polyphenols without organic solvents. These data will be presented and analyzed in an upcoming publication about optimizing the extraction of polyphenols from *S. ramosissima* with water as solvent. The extracts and isolation trials produced by Aalborg University are based on Soxhlet extract, and the extracts and isolation trials produced by Celabor are based on DSCE extracts. Using the 25 L Soxhlet at Aalborg University, Denmark, and the DSCE at Celabor, Belgium, the compositions of polyphenols shown in Table 1 and Table 2 were obtained.

### 2.2. Membrane Filtration

#### 2.2.1. Sequential Membrane Filtration

**Figure 1:**
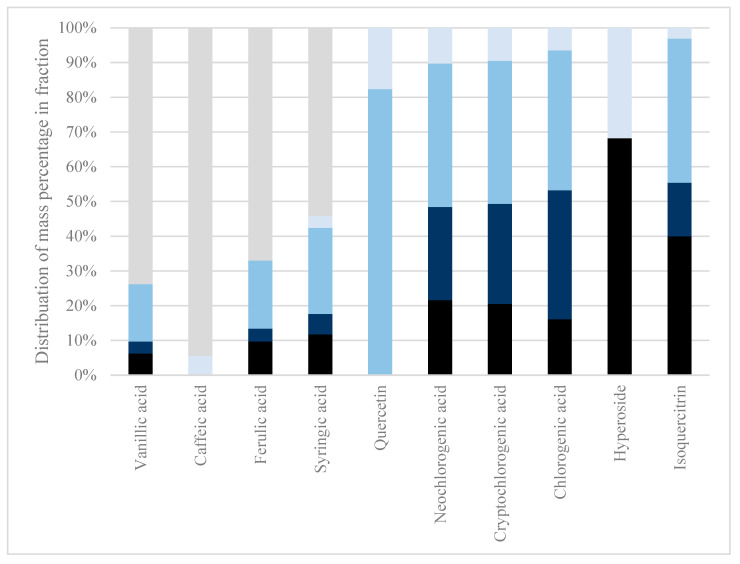
Sequential membrane filtration. The retained fractions after each membrane filtration in a sequential membrane filtration fractionation were analyzed for their polyphenol content. The analytes protocatechuic acid, p-coumaric acid, syringic acid, isorhamnetin, and astragalin were below quantification level on LC-MS and, therefore, not presented. Black: 300 kDa retentate. Dark blue: 100 kDa retentate. Medium blue: 10 kDa retentate. Light blue: 1 kDa retentate. Grey: 1 kDa permeate.

Sequential membrane filtration was conducted to investigate the retention of polyphenols in the different membrane MWCO values and possibly understand macro molecular interactions with polyphenols, e.g., polyphenol–protein interactions, polyphenol–lignocellulosic interactions (see Figure 1).

During conventional membrane filtration at 300–1 kDa, only 55.88 w% DM passed through the membrane as permeate, hence, 44.12 w% DM was retained. Of the permeate, only vanillic acid, caffeic acid, ferulic acid, and syringic acid passed through, with 73.7 w%, 94.5 w%, 67.1 w%, and 54.3 w%, respectively. As only the HCAs and a hydroxybenzoic acid passed through the membranes, all other analytes were found to be retained in higher MWCO membranes. As polyphenols form covalent bonds with proteins, this might be why it was troublesome to use physical isolation methods, such as membrane filtration, to isolate polyphenols from other phytochemicals without prior chemical treatments to the extract. Certain less polar solvents could interrupt polyphenol–protein covalent bonds by changing solvent systems, as indicated by the review of Ozdal et al. [24].

As the permeate has a neutral pH after the first membrane filtration, there is no charge on the membrane to repel protonated polyphenols; hence, the filtration should be sufficient, but still, as seen in Figure 1, there is a large quantity of the individual polyphenols shown to be stuck in the retentate. This could be due to the interaction between the high MW polymeric lignocellulose or protein and the polyphenols. The interaction between polyphenols and other compounds or polymers can be investigated by performing acid hydrolysis on the extract followed by membrane filtration. As the polyphenols of interest are acids that protonate and stabilize at pH < pKa conditions, acid hydrolysis can break the potential interactions between polymeric lignocellulose and the polyphenols of interest.

#### 2.2.2. Acid Hydrolysis Membrane Filtration

After hydrolyzing the lignocellulosic structure by acid hydrolysis and neutralization using Ba(OH)_2_, the first membrane used was a 100 kDa membrane. The filtration was much faster than the sequential membrane filtration and did not leave any retentate apart from an insignificant amount of fouling on the membrane. The filtration took around 30 min for 1000 mL. The color of the permeate was light orange after filtration. The second filtration on the permeate was conducted using the 1 kDa membrane. The membrane filtration using 1 kDa was also fast and did not leave any retentate apart from an insignificant amount of fouling on the membrane. The filtration took around 1 h for 1000 mL. The color after filtration was light yellow. This led to a filtration with a final membrane of 150 Da. The smallest molecule analyzed by UPLC-TQMS was protocatechuic acid, with a monoisotopic mass of 154.03 Da, meaning a membrane with a MWCO 150 Da membrane size would, if the MWCO is at exactly 150 Da, retain all compounds analyzed by UPLC-TQMS. The filtration took approximately 5 h for 1000 mL. The color and pH of the permeate after membrane filtration were clear, transparent, and pH 3, respectively. As the acidity of the permeate was maintained at pH 3, this indicated that the sulfuric acid passed through the membrane, as both H^+^ and SO_4_^2−^ in the solution are smaller than 150 Da.

**Figure 2:**
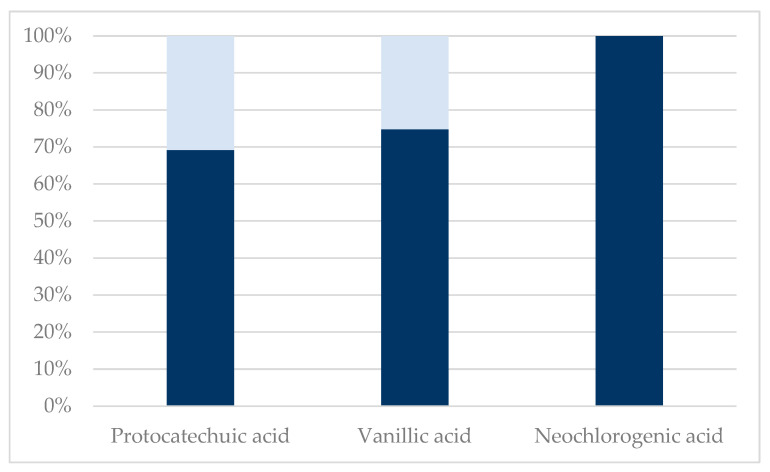
Sequential membrane filtration using 100 kDa, 1 kDa, and 150 Da membranes after acid hydrolysis. The analytes p-coumaric acid, caffeic acid, ferulic acid, syringic acid, quercetin, isorhamnetin, cryptochlorogenic acid, chlorogenic acid, astragalin, hyperoside, and isoquercitrin were below quantification level on LC-MS, and therefore not presented. Dark blue: 150 Da retentate. Light blue: 150 Da permeate.

Due to an immeasurable amount of retentate after the 100 kDa and 1 kDa membranes, the DM% could not be calculated. The low decrease in total polyphenol content with decreasing membrane MWCO size indicates near-zero retention of polyphenols in the membranes and negligible degradation of polyphenols measured by the Folin–Ciocalteu assay by the acid pretreatment. Therefore, these results indicate that the polyphenols of interest in the aqueous extract prepared by Soxhlet from *S. ramosissima* are bound in large molecular complexes with compounds that can be hydrolyzed in a hot 4% H_2_SO_4_ solution, and this bond can thus be destroyed using acid hydrolysis, as seen in Figure 1 and Figure 2.

It is, however, worth mentioning that flavonoid glycosides, i.e., isoquercitrin and hyperoside, were neither present in the permeate nor retentate obtained from membrane filtration of the acid-hydrolyzed extract. This clearly indicates a deglycosylation and further degradation of the aglycones, as quercetin was also not present in the obtained permeate and retentate fractions. Furthermore, an increase in concentration of hydroxybenzoic acids, protocatechuic acid, and vanillic acid (see Table 1), also indicates the degradation of larger polyphenols to hydroxybenzoic acids or the liberation of hydroxybenzoic acids from the lignocellulosic matrix.

Wang et al. stated that the ether groups of glycosylated quercetins, taking the form of rutin and isoquercitrin, are easily broken by 0.5% sulfuric acid at 70 °C for 20 h, yielding 1.25% isoquercitrin and 2.57% quercetin from pure rutin [32]. These acidified conditions used in this study are with higher acid concentrations and temperatures, which could increase the cleavage of ether groups in many polyphenols and their glycosylated derivatives.

The DM that permeated the initial membranes was much lower (61.4%) than the initial amount added to the extract prior to acid hydrolysis. This can be explained by membrane fouling, possible co-precipitation of particles with BaSO_4_, and the degradation of organic compounds due to acid hydrolysis, to CO_2_, and other volatile gaseous compounds like furanic compounds.

### 2.3. Liquid–Liquid Extraction

The necessity of acidifying an extract to eliminate the ionic derivates of the polyphenols was investigated. To increase the extraction yield, the pH should be lower than the pKa of a compound, as this will ensure the compounds are stable and have the highest possible transfer into the EtOAc phase. Ferulic acid has a pKa of 3.6, meaning it is in chemical equilibrium with its ion, ferulate, at pH 3.6. As ferulate has a larger dipole moment than ferulic acid, this ion will have a lower partition coefficient towards EtOAc than ferulic acid in a two-phase liquid–liquid extraction. A liquid–liquid cascade extraction system was set up with sequential EtOAc and n-BuOH liquid–liquid extractions. If the polyphenols of interest are charged ions, their water-solubility is higher, making the liquid–liquid extraction less effective.

**Figure 3:**
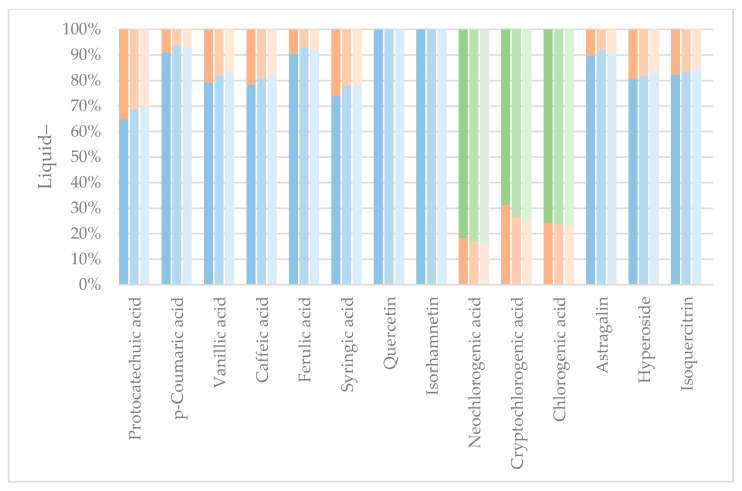
Protonated liquid–liquid extraction and the distribution of individual polyphenols. Darkest color: pH 7, medium color: pH 5, lightest color: pH 3. Blue colors: EtOAc fraction. Orange colors: BuOH fraction. Green colors: Remaining water fraction.

Three liquid–liquid extractions were performed (see Figure 3), and the distribution of the polyphenols was calculated in the sequential liquid–liquid extractions. Similar extraction results were obtained regardless, though a tendency was found that protonated compounds at lower pH would be isolated better in the lower polarity solvent, EtOAc, especially the phenolcarboxylic acids.

**Figure 4:**
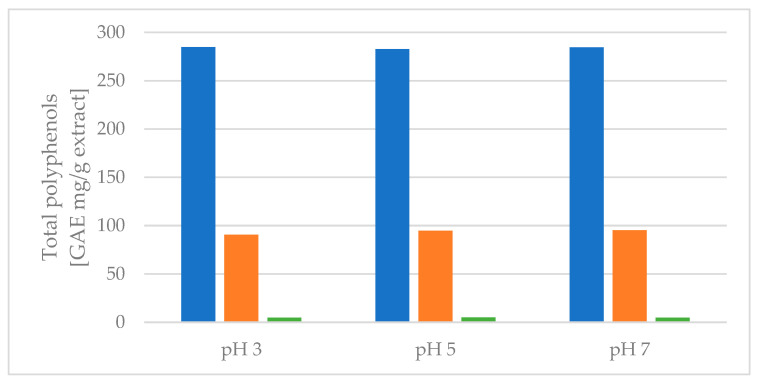
Protonated liquid–liquid extraction and the distribution of total polyphenols extracted, measured by the Folin–Ciocalteu assay. Blue colors: EtOAc fraction. Orange colors: BuOH fraction. Green colors: remaining water fraction.

As seen from Figure 4, no change was observed in the amount of total polyphenols extracted when the pH was changed. This result indicates that the polyphenols are not degrading within the pH range of 3–7, and protonation or deprotonation does not influence the measurement using the assay.

EtOAc generally showed good extraction efficiency, and the second extraction of BuOH, regardless of the pH level, extracted the remaining polyphenols of interest despite the chlorogenic acids. As the partitioning coefficients (LogPs) of the chlorogenic acids are negative, making them more likely to end in the water fraction in octanol:water liquid–liquid extraction, these results correspond well with the theory. As octanol has a higher relative polarity than EtOAc, this indicates that the LogP values of chlorogenic acids are even lower for an EtOAc:water liquid–liquid extraction than octanol:water liquid–liquid extraction, also explaining the low mass transfer of chlorogenic acids from water to EtOAc. In fact, the chlorogenic acids seemed to increase polarity when protonated at lower pH, which could indicate that the initial deprotonation at increasing pH happened at a polar site of the molecule. Results from Figure 3 and Figure 4 show that the effect of protonation of the compounds of interest to increase polarity, and thus affinity towards EtOAc in a liquid–liquid system, is present, but the effect is perceived to be minor. Liquid–liquid extraction was, regardless of pH, shown to be a good method for full isolation of the flavonoid aglycones quercetin and isorhamnetin from the aqueous extract without extraction of chlorogenic acids. This could be used as a primary separation method of flavonoids and chlorogenic acids.

### 2.4. Dynamic Resin Adsorption

**Figure 5:**
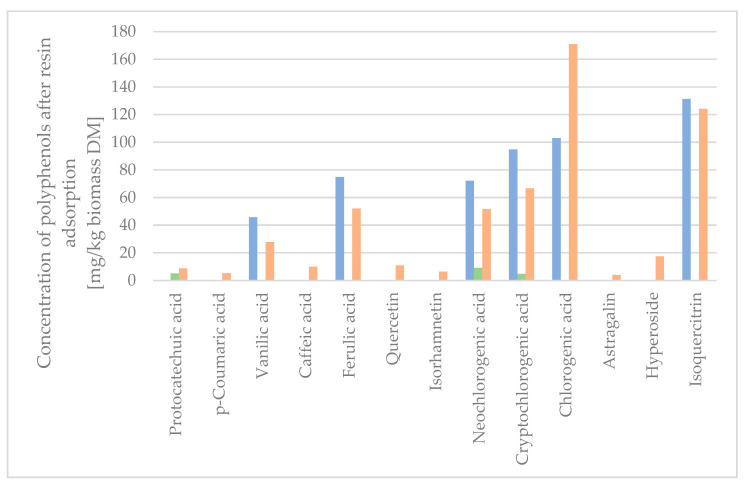
Concentration of polyphenols after XAD-4 resin adsorption. Syringic acid was below quantification level on LC-MS, and therefore not presented. Blue: Raw dry extract. Green: Wash water from XAD-4 resin adsorption. Orange: Desorbate from XAD-4 resin adsorption.

XAD-4 resin eluted by EtOH was compared to the extract produced by DSCE. The quantification of the polyphenols by UPLC-TQMS in the different fractions was calculated as the quantity extracted per mass unit of raw material treated with DSCE in Figure 5.

As the quantity of protocatechuic acid, p-coumaric acid, caffeic acid, quercetin, isorhamnetin, astragalin, and hyperoside was low in the initial extract sample, the concentration was calculated to be below the quantitation limit, and hence not shown in Figure 5. The quantifiable amount of the compounds of the XAD-4 adsorptions was higher than the quantifiable amount of compounds from the raw extract, as the concentration of the compounds of interest was higher in the fractions obtained by resin adsorption. The sum of the compounds isolated, when calculated in mg/kg biomass DM, was 106.5 w% for the XAD-4 resin desorbate with 3.6 w% lost in the water washing the column post-adsorption. The concentration of the extract on the XAD-4 resin resulted in a minimal loss of active compounds in the washing waters, which were found to be mainly chlorogenic acids due to the lower affinity towards XAD-4 resin. Seven polyphenols were completely adsorbed on the XAD-4 resin (p-coumaric acid, quercetin, isorhamnetin, chlorogenic acid, astragalin, hyperoside, and isoquercitrin), despite some of the analytes containing polar and semi-polar functional groups (glucose, galactose, carboxyl group, and quinic acid).

To determine the concentration factor, the wash water and desorbate were freeze-dried and measured for the mass of individual polyphenols per mass unit of the dry extract. As seen in Table 2, the concentration of the total amount of analytes in the eluted dry fraction was 20,204 µg/g, equivalent to 2.020 w%, compared to the dry raw extract, which had a concentration of 0.235 w%. This was an 8.6-fold increase in the total concentration of polyphenol analytes. The mass recovery yield of the concentration step was 9%, and the total recovery of the targeted compounds was between 80 and 100%.

### 2.5. Centrifugal Partition Chromatography

Two CPC fractionations were performed; the first used the descending mode, and the second used the ascending mode. For each of the runs, the fractionation time was 60 min, and one fraction was collected per minute. The first six fractions were discarded for each run, as these corresponded to the CPC column dead volume. To determine the composition of targeted compounds, the 60 collected fractions were screened by UPLC-TQMS, except the first 6 fractions. Of the descending mode CPC fractionation, the 54 fractions were pooled into 7 fractions (P1–P7) according to the identified compounds by LC-MS analysis in each of the 54 sub-fractions and a fraction containing DM, but none of the analytes (P8). Of the ascending mode CPC fractionation, 54 fractions were pooled into 7 fractions (NP1–NP7) and a fraction containing DM, but none of the analytes (NP8). After pooling and freeze-drying, the resulting fractions were analyzed by UPLC-TQMS to quantify the targeted compounds in the different fractions, and the resulting concentrations of analytes in the fractions were calculated, as shown in Table 3.

P1 yielded a good recovery of a mixture of the three chlorogenic acid forms, though in low concentration, indicating a co-isolation of multiple other compounds, P2 showed good recovery of isoquercitrin and hyperoside, P3 recovered the astragalin in a low resulting concentration, P4 recovered a very high concentration of protocatechuic acid with 398.67 mg/gDM, P5 recovered quercetin in low concentration, P6 showed recovery of ferulic acid and vanillic acid, and P7 showed recovery of caffeic acid. The ascending mode CPC fractionation had similarly efficiently separated fractions. NP1 contained the majority of the HCAs, NP2 contained protocatechuic acid at a much lower concentration than P4, NP3 contained astragalin in a similar concentration as P3, NP4 contained isoquercitrin and hyperoside in a similar concentration as P2, NP5–NP7 recovered the three forms of chlorogenic acids in separate fractions, and also achieved higher concentrations of the compounds than P1. NP4, containing the flavonoid glycosides isoquercitrin and hyperoside; and NP6, containing cryptochlorogenic acid, were isolated to a high concentration of 155.27 mg/gDM and 101.50 mg/gDM, respectively. The ascending elution mode seems to be more adapted to create a fraction rich in HCAs, another rich in isoquercitrin and hyperoside, and to recover the three chlorogenic acid forms separated into three different fractions. Furthermore, the ascending elution mode gave a fraction containing 14.0% DM with no analytes, against the 2.3% DM with no analytes in the descending elution mode.

## 3. Conclusions

In membrane filtration at 300–1 kDa, 55.88 w% of the DM passed through, retaining 44.12 w% DM. Only vanillic acid, caffeic acid, ferulic acid, and syringic acid crossed all the membrane into the permeate with 73.7 w%, 94.5 w%, 67.1 w%, and 54.3 w% penetration, respectively. These HCAs passed through while other compounds stayed in higher MWCO membranes. The challenge of physically isolating polyphenols from other chemicals may stem from their covalent bonds with proteins, making methods like membrane filtration problematic without prior chemical treatments.

Sequential liquid–liquid extraction showed easy and fast isolation of the most non-polar compounds, quercetin and isorhamnetin, and the following liquid–liquid extraction isolated the remaining polyphenols of interest, apart from the more polar chlorogenic acids. Full isolation of all analytes of interest, including the more polar chlorogenic acids, was achieved using the non-polar resin Amberlite XAD-4. This method might be slower due to the low volumetric flow rate through in the resin, but it is better regarding occupational health and safety if scaled up due to a lower amount of volatile flammable solvents, slightly soluble in water, creating a significant amount of aqueous waste.

A final fractionation using an ascending elution mode CPC fractionation after resin adsorption yielded relatively pure fractions. High-value and high-bioactivity fractions containing 155.27 mg/g isoquercetin and hyperoside and 101.50 mg/g cryptochlorogenic acid were obtained, with the potential for further purification using CPC in descending elution mode.

This study completed an isolation and fractionation of the polyphenols from *S. ramosissima* for the potential use of polyphenols in food, feed, and pharmaceutical applications.

## 4. Materials and Methods

### 4.1. Raw Material and Chemicals

Lignified biomass of *S. ramosissima* cultivated in an open greenhouse by seawater irrigation by Riasearch, Portugal (40.73835,-8.66183) in October 2019 was used. The biomass used for Soxhlet extraction was rinsed to remove impurities, dried at room temperature, shredded by an agricultural straw shredder (AM55, Agerskov, Denmark) to <2 cm, and washed using a bath of water at room temperature to remove salts. Biomass used for DSCE was washed with water, dried at room temperature, and ground using a knife mill. Biomass was stored dry and dark at room temperature. Dry matter (DM) content was determined by drying the biomass at 105 °C.

The analytical grade chemicals used for the isolation of polyphenols and analysis were ethyl acetate (EtOAc), n-butanol (n-BuOH), ethanol (EtOH), methanol, sulfuric acid, hydrochloric acid (HCl), sodium carbonate, hexane, gallic acid, Folin–Ciocalteu reagent (VWR, Søborg, Denmark), and ultrapure water produced from the Synergy Water Purification System (Merck KgaA, Darmstadt, Germany). Chemicals of LC-MS grade for calibration of the UPLC-TQMS were acetonitrile (VWR, Leuven, Belgium); methanol (Chem-Lab, Zedelgem, Belgium); and formic acid, ammonium formate (Merck KgaA, Darmstadt, Germany). The standard compounds used for the calibration of the UPLC-TQMS were purchased from Sigma-Aldrich (Merck KgaA, Darmstadt, Germany).

### 4.2. Extraction of Polyphenols

Extracts produced for membrane filtration and liquid–liquid extraction were performed using Soxhlet extraction, and extracts produced for resin adsorption and centrifugal partition chromatography were produced using DSCE. Aqueous extracts were spray-dried using an inlet temperature of 130 °C and an outlet temperature of 80 °C by a Mobile Minor spray dryer (GEA Process Engineering A/S, Søborg, Denmark).

#### 4.2.1. Soxhlet Extraction

A quantity of 1770 gDM shredded *S. ramosissima*, sifted using a 2 mm screen to avoid small particles potentially clogging the extraction system, was added to a 12 L Soxhlet basket made from perforated steel. The biomass was subject to extraction in a custom-built 25 L solvent tank pilot-scale Soxhlet (Nordic Engineering, Roskilde, Denmark) using de-ionized water for 8 h, including heating to 100 °C and active cooling, and stirring with approximately 250 rpm. After the extraction, cooling of the equipment was turned on with continuous stirring at approximately 250 rpm for 25 min, and the biomass and extract could be removed. The extract volume was approximately 20 L after each extraction, as the biomass contained absorbed water.

#### 4.2.2. Dynamic Sub-Critical Water Extraction

A quantity of 800 gDM ground *S. ramosissima* was inserted into a DSCE sample container of 6 L, and water was heated to 140 °C in a 20 L reactor (SEPAREX, Champigneulles, France). In the sample container, the preheated water was percolated through the biomass, and percolation cycles of 30 min were performed at 17 bar. After the extraction, the reactor was depressurized and cooled.

### 4.3. Membrane Filtration

#### 4.3.1. Sequential Membrane Filtration

To investigate the membrane filtration of *S. ramosissima* extract and retention of polyphenols, a set of membranes with four different molecular weight cut-off (MWCO) sizes was chosen. TriSep membranes (Lenntech, Delfgauw, The Netherlands) were chosen for this study in the MWCO range of 300, 100, 10, and 1 kDa, with a working pH range of 2–11. A quantity of 20 gDM Soxhlet extract was dissolved in 1 L water under stirring for 10 min. The liquid extract was centrifuged for 10 min at 4700 rpm. The supernatant from the centrifugation was decanted, and the volume was measured. The supernatant was decanted into the membrane feed reservoir and pumped through a cross-flow filtration system. The volumes of the retentate and the permeate were measured when the volumetric flow rate of the feed dropped below one drop per 5 s, indicating a high amount of fouling on the membrane, decreasing the permeate flux. The membrane filtrations were carried out consecutively, with the membrane with the largest MWCO first and the membranes with decreasing MWCO later. After each filtration, the permeate was diluted with de-ionized water to yield 1 L and approximately pH 7. The retentate fractions were freeze-dried and analyzed for their total polyphenol content according to the Folin–Ciocalteu assay and specific polyphenols quantified by UPLC-TQMS.

#### 4.3.2. Acid Hydrolysis Membrane Filtration

Acid hydrolysis was performed using a modified protocol on acid hydrolysis by NREL on an extract to eliminate the effect of matrix interference from polyphenol–protein interactions [33,34]. A quantity of 10 g freeze-dried extract was dissolved in 250 mL water and acidified using 250 mL 8 *w*/*w* % H_2_SO_4_. This solution was transferred to a 1000 mL bluecap bottle, inverted to mix, and autoclaved at 121 °C for 10 min. Ba(OH)_2_ was used to neutralize the acidic solution to form the chemically inert salt barium sulfate, BaSO_4_, which is insoluble in water up to around 2.8 mg/L. During the slow neutralization, the viscosity of the liquid increased, so the 500 mL liquid was diluted to 1000 mL by de-ionized water to allow for magnetic stirring, and the neutralization was stopped at pH 3. After neutralization, the solution was centrifuged at 4700 rpm for 10 min to sediment the insoluble salts and suspended particles. The supernatant from the centrifugation, thought to consist of primarily water, H_2_SO_4_, monomeric sugars, degraded metabolic products, minerals/salts/metals (ash), and individual polyphenols, was filtered through 100 kDa, 1 kDa, and 150 Da TriSep membranes (Lenntech, Delfgauw, The Netherlands) at their respective pressure requirements. The retentate and samples of permeate fractions were freeze-dried and analyzed for their total polyphenol content according to the Folin–Ciocalteu protocol and specific polyphenols quantification by UPLC-TQMS.

### 4.4. Liquid–Liquid Extraction

As EtOAc and n-BuOH have been shown to be suitable solvents for extracting polyphenols of *S. ramosissima* by Surget et al., the importance of protonation of the polyphenols is investigated [8]. To investigate polyphenols’ protonation, batches of water were acidified to pH 7, 5, and 3, and the freeze-dried extract was added to yield a DM loading of 3.3 *w*/*v*%. The pH should be lower than the pKa of a compound, as this will ensure the compounds are stable and have the highest possible transfer into the EtOAc phase. Ferulic acid has a pKa of 3.6, meaning it is in chemical equilibrium with its ion, ferulate, at pH 3.6. As ferulate has a larger dipole moment than ferulic acid, this ion will have a lower partition coefficient towards EtOAc than ferulic acid in a two-phase liquid–liquid extraction. A volume of 100 mL of each of the three extracts (pH 7, 5, 3) was added to an Erlenmeyer flask, and 100 mL of EtOAc was added. A magnetic stirring bar was added to the mixture, and the mixture was stirred for 10 min. After liquid–liquid extraction, the biphasic solution was added to a separatory funnel, and the two phases were separated. An emulsion formed between the two clear phases, which was removed separately and centrifuged at 4700 rpm for 10 min. Following centrifugation, two distinct layers were observed, carefully separated, and transferred to their respective fractions. To extract the desired components further, a secondary liquid–liquid extraction was performed utilizing n-BuOH. Once this process was completed, all fractions were subjected to rotary evaporation and freeze-drying to remove any remaining solvents. The weights of the dried fractions and the initial extract were measured to determine their total polyphenol content using the Folin–Ciocalteu protocol, followed by specific polyphenol quantification using UPLC-TQMS.

### 4.5. Dynamic Resin Adsorption

A volume of 3 L of aqueous *S. ramosissima* extract prepared by DSCE was acidified to pH 3 using 38% HCl and poured on a 230 mL column of Amberlite XAD-4 previously washed and activated with acidified water and EtOH. The volume for adsorption used was 13 bed volumes (BV). The treated non-adsorbed extract was collected as raffinate. The column was washed with acidified water at pH 3 until the conductivity dropped under 0.2 mS·cm to indicate that all previously entrapped compounds were removed from the resin matrix. After that, the adsorbed material was desorbed using EtOH. The EtOH fraction was evaporated under reduced pressure and freeze-dried to investigate the retention of polyphenols on the resin matrix. The dried weight of the different fractions and the initial extract were measured in their total polyphenol content according to the Folin–Ciocalteu protocol and specific polyphenol quantification by UPLC-TQMS.

### 4.6. Centrifugal Partitioning Chromatography

Centrifugal partition chromatography (CPC) fractionations were performed on a CPC-250 system (Armen instruments, Vannes, France) using an Arizona two-phase liquid system, as described by Berthod et al., using hexane/EtOAc in a 1/6 (*v*/*v*) ratio as non-polar fraction, and methanol/water in a 1/6 (*v*/*v*) ratio as a polar fraction [35]. This mixture was equilibrated to yield two stable phases, and the resulting phases were separated. The extract used for this fractionation was resin-adsorbed extract produced by DSCE. In the first fractionation, the polar phase was used as the mobile phase and the non-polar phase was used as stationary phase (descending mode). In the second fractionation, the non-polar phase was used as the mobile phase, and the polar phase was used as the stationary phase (ascending mode). The system comprised 800 twin cells for a total volume of 250 mL. A volume of 500 mg of the extract was dissolved in the mobile phase solvent for each fractionation and injected into the CPC. The speed of the rotor was set to 2000 rpm, and the elution rate was 15 mL/min. The equilibrium pressure was 45 bars. A UV-visible diode array detector followed the elution. Volumes of 15 mL fractions were collected in 50 vials for 50 min, after which the stationary phase was recovered in reverse mode for 10 min post-fractionation. Each fraction was freeze-dried and analyzed on the UPLC-TQMS.

### 4.7. Folin–Ciocalteu Assay

The total polyphenol content was determined using the Folin–Ciocalteu assay described by Singleton et al. [36] with minor modifications. A quantity of 25 mg freeze-dried sample was dissolved in 25 mL water using 10 min of sonication and centrifuged at 5000 rpm for 10 min. A calibration curve was made with five calibration concentrations from 25 to 400 mg/L of gallic acid solutions in water. A volume of 0.1 mL of calibration or sample solution was mixed with 0.4 mL Folin–Ciocalteu reagent, 0.6 mL aqueous Na_2_CO_3_ 200 g/L, and 3.9 mL water in 15 mL centrifugal tubes shortly vortex mixed. After 2 h of dark incubation at room temperature, the absorbance was measured at 760 nm by a Genesys 150 spectrophotometer (Thermo Scientific, Waltham, MA, USA). The polyphenol content was expressed as gallic acid equivalent (GAE) milligrams per gDM biomass.

### 4.8. LC-MS Analysis of Extracts

The identification and quantification of specific polyphenols were carried out by a UPLC coupled with a triple quadrupole mass spectrometer (TQ-MS). The analysis was performed as described by Merten et al. with modifications [37].

The setup comprised a Waters UPLC system composed of an Acquity BEH Shield RP 18 column (100 × 2.1 mm internal diameter and 1.7 μm particle size), a quaternary pump, an autosampler, a UV-visible detector, and Xevo-TQ-MS (Waters, Milford, MA, USA). Solid powdered extracts were dissolved with a ratio of 50 mg in 10 mL of 1:1 (*v*/*v*) MeOH:water containing 0.05% formic acid using 10 min of sonication, and the sample was passed through a 0.22 µm poly(tetrafluoroethylene) (PTFE) filter. The LC-MS parameters were as follows: column temperature fixed at 40 °C, sample vials maintained at 13 °C, injection volume of 3.5 µL, mobile phase flow rate of 500 µL/min, desolvation using nitrogen gas at 800 L/h and 550 °C, cone voltage of 3 kV, electrospray ionization (ESI) in negative mode. ESI ionized 14 compounds of interest, and were identified from their retention times, *m*/*z* values, and fragmentation patterns. Two fragments were found for each polyphenol analyte to verify its detection. Concentrations of the polyphenol analytes were calculated using the fragments’ chromatographic areas, with the areas obtained by the fragments of the calibration set standards having concentrations between 0.25 and 10 mg/L. The mobile phase was composed of two solvents: water with 6 g/L formic acid and 126 mg/L as modifiers (A), and acetonitrile with no modifiers (B).

The analysis was run in negative ESI mode gradient system: 0 min 5% B; 0–7.2 min, to 20% B; 7.2–10.0 min, to 25% B; 10–11.5 min, to 50% B; 11.5–12.0 min, to 100% B; 12.0–12.5 min, 100% B; 12.5–14.0 min, to 5% B; 14.0–16.5 min, 5% B.

## Figures and Tables

**Table 1:** Polyphenols quantified by ULPC-TQMS in Soxhlet and the acid hydrolyzed extract of *S. ramosissima*. ND: No data. <QL: below quantification level for UPLC-TQMS.

µg/g of Dry Extract	Soxhlet Extract	Acid Hydrolyzed Extract
Protocatechuic acid	<QL	56
p-Coumaric acid	<QL	ND
Vanillic acid	171	413
Caffeic acid	<QL	<QL
Ferulic acid	127	<QL
Syringic acid	ND	ND
Quercetin	ND	ND
Isorhamnetin	ND	ND
Neochlorogenic acid	127	57
Cryptochlorogenic acid	105	<QL
Chlorogenic acid	99	<QL
Astragalin	<QL	ND
Hyperoside	33	ND
Isoquercitrin	351	ND
Total	1013	526

**Table 2:** Polyphenols quantified by ULPC-TQMS in DSCE extract of lignified *S. ramosissima*, in the acidified water used for washing, and in the concentrated extract, desorbate, after elution. ND: No data, <QL: below quantification level for UPLC-TQMS.

µg/g of Dry Extract	DSCE Extract	Washing	Desorbate	Concentration Factor
Protocatechuic acid	130	98	1196	920%
p-Coumaric acid	<QL	ND	270	ND
Vanillic acid	236	<QL	1744	739%
Caffeic acid	53	<QL	649	1225%
Ferulic acid	267	ND	1631	611%
Syringic acid	ND	ND	ND	ND
Quercetin	<QL	ND	184	ND
Isorhamnetin	<QL	ND	111	ND
Neochlorogenic acid	226	123	2186	967%
Cryptochlorogenic acid	214	96	2030	949%
Chlorogenic acid	228	ND	2395	1050%
Astragalin	<QL	ND	199	ND
Hyperoside	128	ND	1013	791%
Isoquercitrin	870	ND	6595	758%
Total	2352	317	20,204	859%

**Table 3:** Mass transfer of specific compounds of the injected extract after CPC fractionation and the resulting concentration of specified polyphenols in the fractions. HCA: Hydroxycinnamic acids. ND: No data.

Fraction	Polyphenols	DM of Injected Mass in Fraction (%)	Concentration in Fraction (mg/gDM)
Descending mode			
P1	Chlorogenic acids	63.2	10.46
P2	Isoquercitrin and hyperoside	5.5	138.33
P3	Astragalin	5.7	3.49
P4	Protocatechuic acid	0.3	398.67
P5	Quercetin and isorhamnetin	10.6	2.78
P6	Ferulic, syringic, and vanillic acid	9.2	36.68
P7	Caffeic acid	3.2	28.22
P8	Non-analyte DM	2.3	ND
Ascending mode			
NP1	HCAs, quercetin, and isorhamnetin	15.7	29.23
NP2	Protocatechuic acid	9.1	13.14
NP3	Astragalin	7.2	2.76
NP4	Isoquercitrin and hyperoside	4.9	155.27
NP5	Neochlorogenic acid	44.3	4.93
NP6	Cryptochlorogenic acid	2.0	101.50
NP7	Chlorogenic acid	2.8	85.54
NP8	Non-analyte DM	14.0	ND

## Data Availability

The data presented in this study are available.
